# Entrapment neuropathies: a contemporary approach to pathophysiology, clinical assessment, and management

**DOI:** 10.1097/PR9.0000000000000829

**Published:** 2020-07-22

**Authors:** Annina B. Schmid, Joel Fundaun, Brigitte Tampin

**Affiliations:** a Nuffield Department of Clinical Neurosciences, Oxford University, Oxford, United Kingdom; b High Country Physical Therapy, Laramie, WY, USA; c Department of Physiotherapy, Sir Charles Gairdner Hospital, Perth, Western Australia, Australia; d School of Physiotherapy and Exercise Science, Curtin University, Western Australia, Australia; e Faculty of Business Management and Social Sciences, Hochschule Osnabrück, University of Applied Sciences, Osnabrück, Germany

**Keywords:** Entrapment neuropathies, Compression neuropathies, Carpal tunnel syndrome, Sciatica, Radiculopathy, Radicular pain, Pathomechanisms, Assessment, Diagnosis, Management, Treatment

## Abstract

Entrapment neuropathies such as carpal tunnel syndrome, radiculopathies, or radicular pain are the most common peripheral neuropathies and also the most common cause for neuropathic pain. Despite their high prevalence, they often remain challenging to diagnose and manage in a clinical setting. Summarising the evidence from both preclinical and clinical studies, this review provides an update on the aetiology and pathophysiology of entrapment neuropathies. Potential mechanisms are put in perspective with clinical findings. The contemporary assessment is discussed and diagnostic pitfalls highlighted. The evidence for the noninvasive and surgical management of common entrapment neuropathies is summarised and future areas of research are identified.

## Abstract 2

Entrapment neuropathies are a heterogenous condition reflecting distinct underlying pathomechanisms. A contemporary assessment aimed at identifying potential mechanisms may help target management for these patients.

**Key Points**

The wide variety of pathomechanisms at play in entrapment neuropathy may account for patient heterogeneity.
Contemporary clinical assessment involves a detailed history, clinical examination, and additional tests if required.
Noninvasive management is the first-line treatment for patients with entrapment neuropathies.
Future research is required to determine whether patient stratification can improve outcomes in patients with entrapment neuropathies.

## 1. Introduction

Entrapment neuropathies are caused by compression and/or irritation of peripheral nerves as they travel through narrow anatomical spaces. The most common entrapment neuropathy is carpal tunnel syndrome (CTS) with a lifetime risk of 10%, which increases to a staggering 84% in patients with diabetes.^135^ The second most common entrapment neuropathy is cubital tunnel syndrome.^100^ Another common condition is “sciatica,” with reported prevalence values ranging from 1.6% to 43%.^76^ The striking variation in prevalence has been attributed to the varying definitions of “sciatica.”^76^ Indeed, “sciatica” is an arcane term which, depending on its interpretation, may include somatic referred pain, radicular pain (pain evoked by ectopic discharges from a dorsal root or its ganglion),^19^ and radiculopathy (conduction block along a spinal nerve or its roots manifesting clinically with dermatomal sensory loss, myotomal weakness, or reflex changes).^19,129^ Here, we use “sciatica” as an umbrella term for all 3 conditions, but use the more accurate descriptions (eg, radiculopathy, radicular pain) when studies specifically defined their patient population.

The aetiology of entrapment neuropathies remains largely unknown. They share several risk factors across conditions, such as increased body mass index,^115,134^ occupational or physical factors,^78,115^ and predisposing systemic diseases such as diabetes or hypothyroidism.^115,118^ Recently, genetic predisposition is emerging as one of the strongest risk factors for entrapment neuropathies.^2,82,86,165^ In addition to rare variants,^158^ genome-wide association studies have identified several genetic susceptibility loci for entrapment neuropathies.^2,82,86,165^ Intriguingly, many of the genes are related to connective tissue and extracellular matrix architecture. It currently remains unclear whether these genes increase vulnerability by altering the nerve itself (as a substantial proportion consists of connective tissue) or the environment through which the nerve travels (eg, the tunnels).^165^

Over the past decade, significant advances have been made in our understanding of the pathophysiology of entrapment neuropathies and their assessment and management.

This article summarises the current knowledge and highlights the challenges faced when diagnosing and managing patients with entrapment neuropathies.

## 2. Pathophysiology

**Figure 1.**
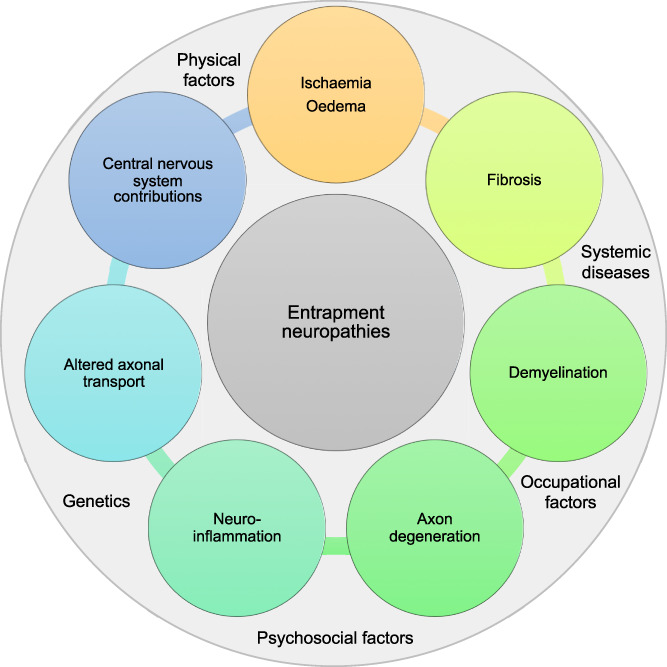
Potential pathomechanisms and risk factors contributing to entrapment neuropathies.

Our understanding of neuropathic pain is largely based on preclinical models involving acute and severe nerve injuries. However, human entrapment neuropathies are quite distinct from these preclinical models because their onset is mostly slow, and the neural injury is often of mild but chronic nature. We^130^ and others^36,62^ have therefore refined animal models that closely mimic human entrapment neuropathies. Below, we summarise the main pathomechanisms that are associated with nerve compression in preclinical and clinical studies (Fig. 1) and provide potential links to the clinical presentation.

### 2.1. Ischaemia, oedema, and intraneural fibrosis

Intraneural ischaemia is typical of mild entrapment neuropathies. Animal models demonstrate that extraneural pressures as low as 20 to 30 mm Hg disrupt intraneural venous circulation.^125^ These pressures are often reached in patients with entrapment neuropathies.^31,60,132,139^ The ensuing reversal of the pressure gradient necessary to assure adequate blood supply could explain the sometimes intermittent paraesthesia, which occur at night, in static positions or with end of range positions (eg, Phalen test for CTS) and may reduce or disappear with movement.

Prolonged ischaemia is likely to induce a compromise of the blood nerve interface^90,170^ with subsequent oedema formation. Clinically, the presence of oedema is apparent by an enlargement of the compressed nerves^103,169^ and increases in signal intensity on specialised magnetic resonance sequences.^32,83,136^ Persistent oedema may eventually lead to intraneural and extraneural fibrotic changes,^92^ which is a feature of both radiculopathies^68^ and entrapment neuropathies of distal nerve trunks.^1,28^ Extraneural fibrotic changes are thought to explain the reduced gliding of human compressed nerves that is apparent during limb movements.^46^

### 2.2. Demyelination and axon degeneration

Prolonged ischaemia and mechanical compromise may induce downstream effects such as demyelination and eventually axon degeneration. Focal demyelination is a hallmark of entrapment neuropathies,^58,63,91,92,107,109,130^ which are often characterised by nerve conduction slowing or block.^95^ Ischaemia in the absence of demyelination may, however, also contribute to nerve conduction changes.^25,73^

Together with demyelination, the architecture of nodes of Ranvier changes after nerve compression,^131^ including both downregulation and upregulation of existing ion channels as well as expression of novel channels.^24,42,49,71,85^ Such changes have been implicated with spontaneous ectopic generation of action potentials,^6,26^ and may contribute to spontaneous electric shock-like pain or symptoms provoked upon Tinel testing.

If nerve compression/irritation perseveres, axons may eventually degenerate. It is commonly believed that entrapment neuropathies predominantly affect the large myelinated fibres (eg, demyelination and axon damage)^36,92^ and that small axons are relatively resistant to compression.^36^ Indeed, large myelinated Aβ-fibre-mediated numbness to light touch is common as is motor neuron dysfunction apparent by myotomal muscle weakness, atrophy, and reflex changes. Guidelines on the diagnosis of entrapment neuropathies such as CTS or cervical radiculopathy thus focus heavily on large fibre tests.^5,20^ Recent evidence, however, suggests that small nerve fibre function and structure are affected in entrapment neuropathies^8,29,50,74,80,126,142,146,155,167^ and may even precede large fibre changes.^9,130,131,141^ These findings suggest that (early) diagnosis of entrapment neuropathies should include tests for small fibre function.

### 2.3. Neuroinflammation

There is a wealth of preclinical data confirming an important role of neuroinflammation in the generation and maintenance of neuropathic pain [for review, see Refs. 99, 151, 163, 166]. Neuroinflammation is characterised by activation of immune cells (eg, macrophages and T-lymphocytes) at the site of damaged axons. Immune cells release inflammatory mediators (eg, cytokines, chemokines, and lipid mediators), which induce a breakdown of the blood–nerve barrier resulting in further immune cell influx and swelling. Importantly, neuroinflammation sensitises injured and uninjured axons and nociceptors in target tissue, contributing to neuropathic pain initiation and maintenance. Whereas most evidence for a link between neuroinflammation and neuropathic pain stems from acute and severe nerve injury models, there is a growing body of evidence suggesting that neuroinflammation is also a feature of mild chronic nerve compression.^61,112,130,133^ Intriguingly though, the neuroinflammation does not remain restricted to the lesion site, but can be found in associated dorsal root ganglia after peripheral nerve compression^130^ or nerve root compromise.^112^ Animal models of radiculopathy also demonstrate an activation of glial cells in the dorsal horn of the spinal cord.^122,123,140^ A similar glial cell reaction has also been reported for severe distal nerve injuries involving substantial axonal damage,^67^ whereas compression injuries with only minimal axonal damage seem insufficient to induce overt spinal cord neuroinflammation.^130^ The presence of local and remote immune inflammation has now been confirmed in patients with lumbar radicular pain using combined positron emission tomography and magnetic resonance imaging (MRI)^3^ The presence of remote neuroinflammation could explain the often observed spread of symptoms beyond affected dermatomes or innervation territories in patients with entrapment neuropathies^23,102,172^ (see https://www.youtube.com/watch?v=BZYtAR4zUpg).

Of note, mild experimental nerve compression can also induce an inflammatory reaction within the epineurium.^130^ Such epineural inflammation may sensitise the nervi nervorum^21^ and induce spontaneous or evoked activity in nociceptive axons resulting in mechanical hypersensitivity despite the absence of frank axonal damage.^40^ Arguably, such extraneural mechanisms may explain why a subgroup of patients with entrapment neuropathies has pain with a predominant nociceptive rather than neuropathic character.

### 2.4. Changes to axonal transport

Peripheral nerve compression^7,33–35,76^ and inflammation^10^ impair retrograde and anterograde axonal transport. Its blockage can result in increased nerve mechanosensitivity,^39^ presumably by the accumulation and insertion of ion channels at the lesion site (see changes to nodal architecture above), and may contribute to symptoms in patients with entrapment neuropathies.

### 2.5. Central nervous system contributions

Because the peripheral and central nervous system form a functional entity, injuries of peripheral nerves inevitably initiate central changes. Central changes after severe experimental nerve injury include, but are not limited to, central immune-inflammatory mechanisms,^81,101,122,123^ central sensitisation,^80^ and changes to cortical representations.^43,104,105,150^ Clinical hallmarks of central mechanisms including bilateral sensory deficits in unilateral painful entrapment neuropathies,^29,37,59,142^ widespread hypersensitivity,^37,53,142,171,172^ and impaired conditioned pain modulation^138^ have been described in patients with entrapment neuropathies. Furthermore, cortical changes have been reported including functional and structural changes of the somatosensory cortex in patients with CTS^43,93,104,150^ and changes in cortical morphometry in patients with lumbar radicular pain.^87^ Clinically, such cortical changes may manifest in an impairment in left/right judgement tasks such as found in patients with CTS.^128^

It is subject of ongoing debate, whether central changes are dependent on peripheral drivers or can drive symptoms independently.^12^ Patients with entrapment neuropathies have continued abnormal input from the peripheral nervous system (too much or too little), which may perpetuate central adaptations. The importance of peripheral drivers in entrapment neuropathies becomes clear from clinical observations of often immediate relief of focal and widespread symptoms after decompression surgery^15,145,155^ or local steroid injections.^97^

### 2.6. Psychosocial factors

Psychosocial factors often play a role as risk factors for the development or persistence of pain. To date, the psychosocial contributions to entrapment neuropathies have not been evaluated in detail. A recent systematic review suggests that there is only limited and inconsistent evidence for a positive association of psychosocial risk factors and CTS.^96^ In patients with “sciatica,” the role of psychosocial features as prognostic factors for conservative management has either not been studied^11^ or remains controversial.^57,77^ Undoubtingly though, entrapment neuropathies can have strong psychosocial consequences as apparent in lumbar radicular pain affecting many aspects of life, including psychological status.^124^ Future work is required to conclusively determine the role of psychosocial factors in the generation, maintenance, and resolution of symptoms in patients with entrapment neuropathies.

## 3. Clinical presentation

The diagnosis and management of these patients can be challenging due to the heterogeneity of pathomechanisms and varied clinical presentation. Interestingly, nerve compression may be asymptomatic, as documented by false positive nerve root compression seen on MRI.^48^ In some patients, nerve entrapment may cause pain in the innervation territory of the affected nerve^55^; however, extradermatomal pain distribution is common (CTS up to 70%^23,172^; radicular pain 64%−70%).^102^ Therefore, clinicians should not rule out entrapment neuropathies in the absence of clearly defined dermatomal/peripheral symptom referral patterns. Nerve entrapment may or may not cause nerve fibre damage; it may affect large and/or small fibres, and the extent of sensory fibre damage may account for heterogeneity of symptoms, ie, sensory loss and/or enhanced pain sensitivity.^131^ The heterogeneity of pathomechanisms likely explains the identification of subgroups of patients with radicular pain^13,94,142^ with differing somatosensory profiles, based on quantitative sensory testing^13,142^ and neuropathic pain screening tools.^94^

The core sign of nerve damage in entrapment neuropathies is loss of function due to reduced action potential conduction caused by the nerve lesion.^146^ Various positive sensory symptoms and signs indicating gain of function have been reported, including paraesthesia, pain attacks, and evoked pain (hyperalgesia and allodynia).^94^ Self-reported pain profiles differ between patients with distal (CTS) vs proximal (radiculopathy) entrapment neuropathies.^146^ Patients with radiculopathy show an increased pain attack severity and pressure, potentially reflecting distinct underlying pain mechanisms. Another feature of gain of function seen in patients with nerve-related pain is heightened nerve mechanosensitivity, which is characterised clinically by pain to mechanical provocation of neural structures (eg, elongation of the affected nerve during straight leg raise testing or nerve compression with manoeuvres such as Spurling test.^4,29,48,108,157^ Historically, neural provocation tests were thought to be diagnostic for entrapment neuropathies; however, heightened mechanosensitivity can occur in the absence of a nerve lesion,^4,143,144,157^ and vice versa, patients with confirmed entrapment neuropathies may present without signs of heightened mechanosensitivity.^14^ Nevertheless, neural provocation tests form an integral part of the clinical examination (see below) as the presence of heightened mechanosensitivity may guide physiotherapeutic management.^127^

Although entrapment neuropathies are considered the most common cause of neuropathic pain, not all patients have obvious signs of nerve damage. For instance, radicular pain from nerve root compression can exist as a discrete disorder without a nerve root lesion and associated loss of function, ie, radiculopathy.^19^ Furthermore, only 30% of patients with cervical radiculopathy are classified as having neuropathic pain based on the painDETECT questionnaire.^144^ However, it has to be acknowledged that neuropathic screening tools were developed before the new definition of neuropathic pain and their validity may be in question.^143,147,148^

## 4. Assessment

### 4.1. Neuropathic pain grading system

**Figure 2.**
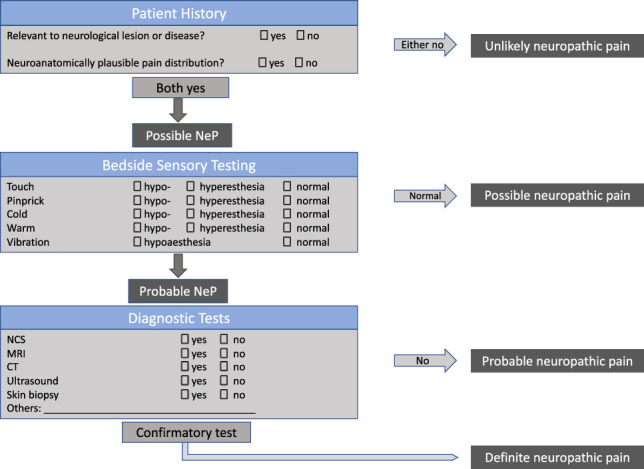
Neuropathic pain grading system adapted from 55.

The Neuropathic Pain Special Interest Group of the International Association of the Study of Pain introduced a grading system to assist clinicians and researchers determining the certainty of neuropathic pain (Fig. 2).^55^ The level of “probable” neuropathic pain is suggested to be sufficient to initiate pharmacological treatment for neuropathic pain.^55^

The application of the grading system may, however, be problematic in patients with entrapment neuropathies because (1) symptoms often spread beyond the affected dermatome or innervation territories of the affected nerve, (2) patients may have very subtle sensory loss that may not be identified with bedside sensory testing and/or patients may present with mainly gain of function, and (3) diagnostic tests may not be indicated (eg, skin biopsies) or may be negative (see imaging and nerve conduction studies below). It therefore often remains unclear whether patients with entrapment neuropathies should receive management according to the neuropathic pain guidelines.^44^

### 4.2. Clinical assessment

The examination of patients with suspected entrapment neuropathies comprises a comprehensive subjective assessment, including the localisation and distribution of symptoms on a body chart as well as their quality, intensity, and behaviour over 24 hours. The medical history provides clues about potential mechanisms of injury or causes leading to pain or neurological lesions and disorder progression. The history may also provide important information on psychological or behavioural factors (orange, yellow flags), social and economic factors (blue flags) as well as occupational factors (black flags) that may contribute to the presentation.^72^ Importantly, clinicians are encouraged to evaluate the presence of differential diagnoses and serious pathologies (red flags) masquerading as entrapment neuropathies (Table 1).^56^

**Table 1:**
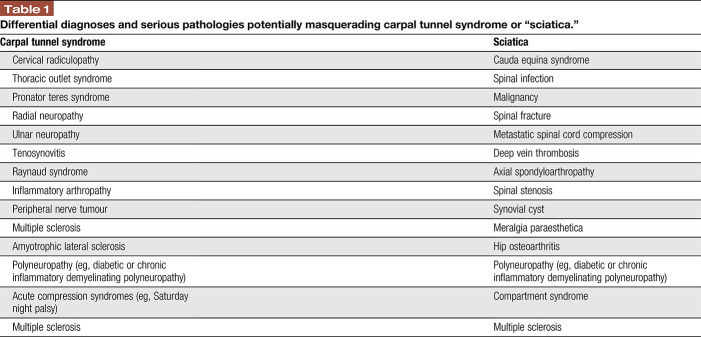
Differential diagnoses and serious pathologies potentially masquerading carpal tunnel syndrome or “sciatica.”

The physical examination includes the assessment of both musculoskeletal and related neural tissue, as well as a detailed neurological bedside examination of sensory and motor function to ascertain the presence of a nerve lesion. Results of medical investigations may assist in the diagnostic work-up. For a detailed discussion on clinical assessment of entrapment neuropathies with a focus on spinally referred pain, please consult the study by Schmid and Tampin.^129^

#### 4.2.1. Neurological examination

**Figure 3.**
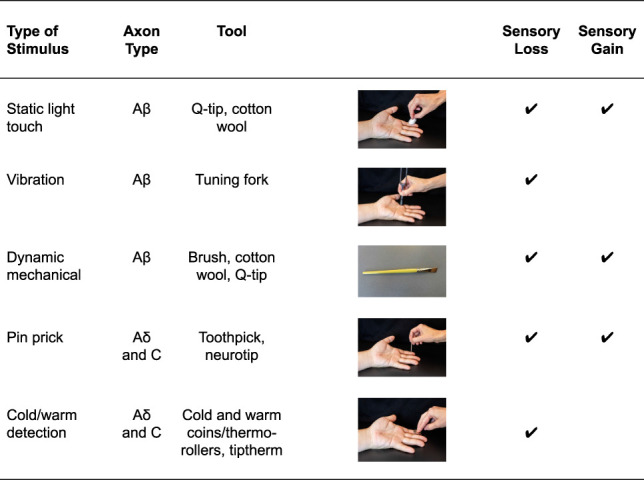
Bedside sensory testing tools.

Motor function assessment comprises the examination of reflex responses and muscle strength testing. Sensory examination must include the assessment of large and small sensory fibres because both can be affected in patients with nerve entrapment.^142,146^ Figure 3 outlines various tools for bedside sensory testing that assess loss and gain of function in distinct sensory fibre populations. The validity of low-cost bedside sensory testing compared to quantitative sensory testing or skin biopsy has been documented in recent studies.^120,173^

#### 4.2.2. Neural mechanosensitivity

Several provocation tests are designed to detect nerve mechanosensitivity such as the Tinel sign or Phalen test for CTS or the Spurling test for cervical radiculopathy. However, the measurement properties of these positional tests vary greatly between studies, indicating uncertainty in their diagnostic utility.^88,153^

The straight leg raise test has been widely used to assess heightened nerve mechanosensitivity in the lower limb.^38,59^ Although the upper-limb analogue (upper-limb neurodynamic test)^47^ is less known in medicine, it is extensively applied in physiotherapy practice.^108^ These tests comprise passive joint movements that cause movement/elongation or tension on neural structures. An abnormal response to the tests is defined as at least partial reproduction of the patient's symptoms with positive structural differentiation.^108^ Structural differentiation uses movements at a site remote to the painful area to further load or unload neural structures, thus differentiating symptoms of neural origin from local musculoskeletal origin.^64^ For detailed description of nerve mechanosensitivity testing, we refer the readers to the studies by Butler,^22^ Schmid and Tampin,^129^ and Tampin.^149^

#### 4.2.3. Imaging and nerve conduction studies

Additional examinations such as MRI, ultrasound, or nerve conduction studies may help in the diagnostic workup. Although nerve conduction tests are particularly useful in the diagnosis of distal entrapment neuropathies (CTS and cubital tunnel syndrome), their value for more proximal neuropathies has not been fully established.^30^ It should be noted that in the early stages of CTS, nerve conduction studies may be unable to detect nerve injury.^131^ MRI is commonly used for proximal entrapment neuropathies (eg, “sciatica”), but often results in false negatives or positives and the affected level identified on imaging and clinical examination often does not match.^159^ Ultrasonography is often preferred over MRI for distal entrapment neuropathies due to its cost-effectiveness, ease of access, and possibility of quick contralateral comparison. The screening for an enlarged nerve diameter might be useful in the diagnosis of CTS^121^ or cubital tunnel syndrome.^27^ In addition to being confirmatory tests, imaging and nerve conduction tests can be important for differential diagnosis and the exclusion of serious pathologies.

## 5. Natural history, prognosis, and management

Due to the scarcity of evidence in less common entrapment neuropathies, we report findings from CTS and “sciatica” only. For ethical reasons, studying the natural history of entrapment neuropathies is challenging. In CTS patients who have not received treatment over an average of 2 years, ∼23% of patients deteriorated, ∼29% remained stable, and ∼48% showed symptom recovery.^111^ A similar pattern presents in patients with “sciatica,” where the clinical course is generally considered favourable; however, at least one-third will develop persistent pain and disability lasting a year or more.^65,69,77,156,161,164^ Unfortunately, prognostic factors for pain persistence remain elusive^11^; the most comprehensive cohort study to date in “sciatica” showed only modest associations with poor outcome for shorter pain duration, belief that symptoms will not last long and overall impact of “sciatica.”^77^ Unexpectedly, the strongest predictive factor (odds ratio 5.62, 95% confidence interval 1.76–17.92) was myotomal weakness, which was, however, associated with a good prognosis. Given the failure or only weak predictive ability of traditional prognostic factors (psychosocial features, MRI, pain severity etc), novel approaches must be considered.

### 5.1. Management

Independent of the type of entrapment neuropathy, clinical guidelines suggest a course of conservative treatment before more invasive options are considered.^20,120,174^

#### 5.1.1. Physiotherapy/occupational therapy for carpal tunnel syndrome

For mild to moderate CTS, therapy usually includes advice, splinting, electrophysiological agents as well as manual therapy and/or exercises.^51^ There is currently limited evidence for a short-term benefit of splinting compared to no treatment.^113^ Splinting is recommended at night only.^51^ A Cochrane review reported that there is limited evidence for a beneficial effect of exercise and mobilisation in the management of patients with CTS.^114^ This was supported by another review suggesting that neural mobilisation did not improve clinical outcomes compared to other treatments.^16^ However, more recent randomised controlled trials suggest a benefit of more comprehensive hand therapy packages.^52,54,84^ Given the limited literature available for the effectiveness of therapeutic management of CTS, the decision to provide physiotherapy/occupational therapy management should be based on the clinicians' expertise and patients' preferences^114^ while following current treatment recommendations.^51^

#### 5.1.2. Physiotherapy for “sciatica”

Physiotherapy is recommended as the first-line treatment for patients with “sciatica.”^106^ Based on studies with mostly high risk of bias and heterogeneity of physiotherapy interventions, we found inadequate evidence to make clinical recommendations on physiotherapy for patients with “sciatica.”^41^ Importantly, “sciatica” definitions in available studies varied significantly and the studied populations were heterogeneous making firm conclusions difficult. There is some indication that distinct subgroups of patients may react better to certain physiotherapy treatments. For instance, patients with heightened neural mechanosensitivity seem to benefit most from neural mobilisations compared to patients with radiculopathy or radicular pain.^127^ Large, well-designed trials are required to determine the most effective physiotherapy approach and whether treatment stratification may improve outcome.

#### 5.1.3. Pharmacological interventions

Oral medications do not seem to be beneficial in patients with CTS, with nonsteroidal anti-inflammatories, diuretics, or vitamin B6 not being superior to placebo.^110^ Local steroid injections seem to have a short-term benefit only, which exceeds the effect of orally taken steroids.^97^ Similar results are reported by several meta-analyses for patients with “sciatica,” suggesting that nonsteroidal anti-inflammatories,^119^ oral corticosteroids,^116^ Tumour necrosis factor blockers,^162^ opioids,^116^ and specific neuropathic pain medications (eg, anticonvulsants and antidepressants) do not provide better symptom relief than placebo.^116^ Again, corticosteroid injections seem to provide a small benefit in the short but not long term.^117^

This disappointing pharmacological efficacy in patients with entrapment neuropathies may in part be due to our limited understanding of the exact pathomechanisms. Importantly, the heterogeneity of patients within one diagnosis may mask potential beneficial effects of specific subgroups. Future work is required to determine whether stratification methods could be used to identify distinct populations that may benefit from specific pharmacological interventions.

#### 5.1.4. Surgery for carpal tunnel syndrome

Carpal tunnel decompression is the most common upper-limb surgery^45^ and surgery rates are predicted to double over the coming decade,^17^ posing a significant challenge to public health systems. The 2 surgical approaches used are endoscopic and open carpal tunnel release, with no strong evidence indicating one technique to be more superior.^160^ Although CTS surgery is usually successful, ∼25% of patients do not benefit.^18^ Potential complications include scar tenderness, persistent symptoms, neurovascular injury, wound complication, and reduced grip strength.^98^ Indications for surgical consultation include moderate-to-severe or deteriorating symptoms, daily symptoms, frequent night waking, persistent symptoms causing functional impairment, not responding to nonsurgical treatments (120), and patients' preference.^152^

#### 5.1.5. Surgery for “sciatica”

Lumbar microdiscectomy is the most common type of surgery performed to relieve nerve root irritation or compression due to a herniated disk. Pooled data of >13,000 patients undergoing surgery for “sciatica” demonstrated that although surgery is followed by a rapid decrease in pain and disability by 3 months, patients still experienced mild-to-moderate pain and disability 5 years after surgery.^89^ Of note, ∼4% of patients are worse after surgery^137^ and reoperation rates of ∼12% within 4 years have been reported after discectomy.^66^ Factors associated with negative postoperative outcomes include intact annulus fibrosus, longer duration of sick-leave, worker's compensation, and greater severity of baseline symptoms.^168^ Two systematic reviews suggest that surgery may yield faster pain relief and perceived recovery than physiotherapy or physical activity for patients with “sciatica;” however, long-term outcomes are comparable.^70^ Given the nonsuperiority of surgery in the long-term and the significant risks and side effects, conservative management should remain the first-line treatment. However, surgery is indicated in the presence of severe or progressive neurological deficits or persistent symptoms that are unresponsive to conservative treatment.^106^ Future research is needed to better understand appropriate patient selection and timelines for surgery to improve outcomes and patient satisfaction.

## 6. Future challenges and conclusion

**Figure 4.**
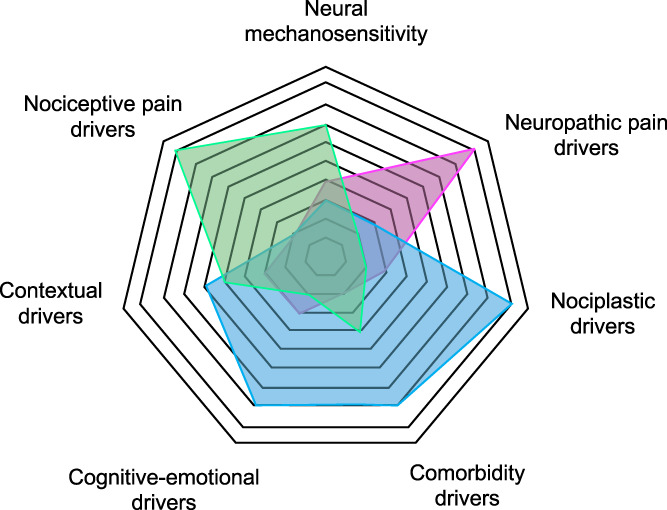
Potential drivers contributing to specific multidimensional profiles in patients with entrapment neuropathies. The spiderweb highlights how distinct drivers may be more prominent in 3 distinct patient presentations (green, blue, and pink). The weighting of these drivers in individual patients may contribute to the design of personalized management for patients with entrapment neuropathies.

The somewhat disappointing evidence of pharmacological, physiotherapeutic and surgical management of entrapment neuropathies clearly highlights that a one-size-fits-all approach is not successful. Considering the distinct pathomechanisms and contributing factors, contemporary management should be personalised, considering the multidimensional profile of an individual patient (eg, different pain mechanisms at play, contextual factors, cognitive emotional drivers, comorbidities, Fig. 4).^154^ As such, individualised management will vary in patients with entrapment neuropathy and should target the predominant dimensions. For instance, tissue-specific interventions (eg, specific exercises, postural modification, mobilisations, targeted pharmacology, injection, and surgery) may be indicated in patients with clear nociceptive, heightened neural mechanosensitivity or neuropathic components. By contrast, multidisciplinary systemic treatments (eg, cognitive behavioural and functional approaches, general exercise) may be more suitable for patients with strong cognitive–emotional, contextual, and nociplastic drivers. Future research is required to further understand specific drivers at play in individual patients, their utility for predicting outcome and treatment stratification, and the effect of personalised treatment on outcomes before a truly evidence-based management of patients with entrapment neuropathies is possible.

## Disclosures

The authors have no conflict of interest to declare.

A.B. Schmid is supported by the National Institute for Health Research (NIHR) Oxford Biomedical Research Centre (BRC). Brigitte Tampin was supported by the Government of Western Australia, Department of Health, and Raine Medical Research Foundation. The views expressed are those of the authors and not necessarily those of the NHS, the NIHR or the Department of Health.
